# Do the antipredator strategies of shared prey mediate intraguild predation and mesopredator suppression?

**DOI:** 10.1002/ece3.2170

**Published:** 2016-05-10

**Authors:** John D. J. Clare, Daniel W. Linden, Eric M. Anderson, David M. MacFarland

**Affiliations:** ^1^ College of Natural ResourcesUniversity of Wisconsin Stevens PointStevens PointWisconsin; ^2^ New York Cooperative Fish and Wildlife Research UnitCollege of Natural ResourcesCornell UniversityIthacaNew York; ^3^ Wisconsin Department of Natural ResourcesRhinelanderWisconsin

**Keywords:** Coyote, ecology of fear, fox, hierarchical abundance models, intraguild predation, mesopredator suppression, white‐tailed deer, wolf

## Abstract

Understanding the conditions that facilitate top predator effects upon mesopredators and prey is critical for predicting where these effects will be significant. Intraguild predation (IGP) and the ecology of fear are hypotheses used to describe the effects of top predators upon mesopredators and prey species, but make different assumptions about organismal space use. The IGP hypothesis predicts that mesopredator resource acquisition and risk are positively correlated, creating a fitness deficit. But if shared prey also avoid a top predator, then mesopredators may not have to choose between risk and reward. Prey life history may be a critical predictor of how shared prey respond to predation and may mediate mesopredator suppression. We used hierarchical models of species distribution and abundance to test expectations of IGP using two separate triangular relationships between a large carnivore, smaller intraguild carnivore, and shared mammalian prey with different life histories. Following IGP, we expected that a larger carnivore would suppress a smaller carnivore if the shared prey species did not spatially avoid the large carnivore at broad scales. If prey were fearful over broad scales, we expected less evidence of mesopredator suppression. We tested these theoretical hypotheses using remote camera detections across a large spatial extent. Lagomorphs did not appear to avoid coyotes, and fox detection probability was lower as coyote abundance increased. In contrast, white‐tailed deer appeared to avoid areas of increased wolf use, and coyote detection probability was not reduced at sites where wolves occurred. These findings suggest that mesopredator suppression by larger carnivores may depend upon the behavior of shared prey, specifically the spatial scale at which they perceive risk. We further discuss how extrinsic environmental factors may contribute to mesopredator suppression.

## Introduction

Evidence increasingly suggests that apex predators can exert disproportionate ecological influence within communities (Ripple et al. 2014). These effects include density‐mediated or trait‐mediated top‐down regulation of prey and mesopredators that may further trickle down to benefit species at lower trophic levels (Palomares and Caro 1999; Ripple and Larson 2000; Berger et al. 2001; Berger and Gese 2007; Berger et al. 2008; Johnson and VanDerWal 2009; Bechsta and Ripple 2009; Prugh et al. 2009). However, the strength of apex predator effects upon prey or mesopredators is heterogeneous (Levi and Wilmers 2012; Newsome and Ripple 2015). Understanding mechanisms or correlates underlying this heterogeneity is critical for predicting top‐predator effects.

Studies of top‐predator ecosystem effects are generally contextualized within two distinct theoretical hypotheses. The intraguild predation hypothesis (IGP hereafter) describes spatial patterns in large and small predator abundance as driven by the consumptive effects a large predator has upon a smaller predator (Holt and Polis 1997). A general assumption of the hypothesis is that the large and small predator share a prey resource that is relatively static in space (Holt and Polis 1997). The hypothesis predicts that a larger predator should competitively exclude a smaller predator from prey‐rich regions while the subordinate species exists in prey‐poor regions incapable of supporting the larger species. The smaller predator may coexist with the larger predator by making use of spatiotemporal refugia, or in areas with moderate levels of prey abundance (Holt and Polis 1997; Amarasekare 2007, 2010; Robinson et al. 2014). Empirical studies have found support for IGP in mammalian carnivore communities, although both consumption and intimidation have been identified as underlying suppressive mechanisms (Cypher et al. 2000; Steinmetz et al. 2013; Robinson et al. 2014; Swanson et al. 2014).

The ‘ecology of fear’ or ‘fear landscapes’ (Brown et al. 1999) are conceptual frameworks that describe how a predator intimidates its prey, effecting the space use of both organisms. The framework assumes that prey and predator are both mobile, and that individual prey balance space‐use between regions of foraging value and regions of foraging risk (Brown et al. 1999; Lima 2002; Mitchell and Lima 2002). When intimidation effects are greater than consumptive effects upon prey species, theoretical expectations are that a predator will spend more time in areas where prey are easily captured but not abundant, while prey primarily congregate in low‐risk space (Laundre 2010).

Ecology of fear and IGP are not mutually exclusive: trade‐offs between risk and resource availability may incur fitness costs to both a prey species or smaller competitor alike. But the hypotheses have incompatible assumptions regarding how prey resources respond to predation, making it difficult to unite the two within a single theoretical framework. In practice, large predators appear to kill and intimidate both smaller predators and prey (Sergio et al. 2003; Creel et al. 2005; Hebblewhite et al. 2005; Preisser et al. 2005). If both a smaller predator and a shared prey species actively avoid a large predator in space, than the smaller predator may no longer be forced to make direct trade‐offs between risk and resource availability. Thus, it seems reasonable that intraguild predation effects mesopredator fitness more in systems where the shared prey items are comparatively sessile than when shared prey perceive a larger predator to be more dangerous and spatially respond to its presence at a large scale.

In this context, prey mobility relates to the intrinsic movement ability of species, but also the scale at which they perceive predation risk and move to avoid it (i.e., a spatial ‘neighborhood of fear’). These characteristics may be highly correlated with prey life‐history (Stearns 1992). Small species with extremely high rates of mortality typically develop fast life histories to swamp predation with reproduction (Promislow and Harvey 1990), and should have a relatively small fear neighborhood. Species with slower life histories generally invest more in behavioral strategies to avoid predation, are typically more prone to flight, and exhibit larger flight distances (Caro 2005; Blumstein 2006). If intraguild predation or mesopredator suppression is indirectly dependent upon the antipredator strategies of shared prey, then it may further be partially dependent upon prey life history.

Our overarching goal was to evaluate patterns in IGP in the context of fear‐based antipredator behaviors and prey life‐history. Our model system primarily focuses on a commonly considered three‐level carnivore community (Levi and Wilmers 2012; Newsome and Ripple 2015) in a mixed‐used landscape: red fox (*Vulpes vulpes*), gray fox (*Urocyon cineargentinus*) coyote (*Canis latrans*), and gray wolf (*C*. *lupus*). Although each species has been identified causing or responding to competitive interactions, there has been little exploration of their relations within the theoretical context of IGP. We considered two prey species with different life‐histories as partially shared resources. White‐tailed deer (*Odoccoileus virginianus*) represent a prey species with high mobility and limited fecundity, traits consistent with hypothetical prey within the ecology of fear hypothesis (Brown et al. 1999; Laundre 2010), and are preyed upon by both coyotes and wolves. Lagomorphs are an important prey item for coyotes and foxes (Bekoff and Gese 2003; Cypher 2003) with fast life histories characterized by high fecundity and limited space‐use. These traits suggest that lagomorphs should behave more like the immobile resource assumed under IGP, likely to perceive and respond to risk at a smaller scale. We considered interactions in relative abundance and detection between two distinct triangular relationships: (1) wolves – coyotes – deer, and (2) coyotes – foxes – lagomorphs.

We expected that associations between wolves and deer would follow the ecology of fear expectations, with wolves using sites of increased risk negatively correlated with deer abundance, and deer using space with lower risk. Despite opposing empirical evidence from other regions (Berger and Gese 2007), we expected that wolves would not suppress coyotes given theoretical expectations and local evidence from similarly fragmented areas (Levi and Wilmers 2012; Newsome and Ripple 2015). In contrast, we expected that coyotes would suppress foxes. Following IGP, we further expected that coyote abundance or individual space use would positively associate with lagomorph abundance, and that fox abundance or individual space use would negatively associate with lagomorph abundance and coyote abundance (Robinson et al. 2014). Our evaluation was based upon remote camera images collected in central Wisconsin, USA using hierarchical models of species distribution and abundance.

## Materials and Methods

### Sampling

**Figure 1:**
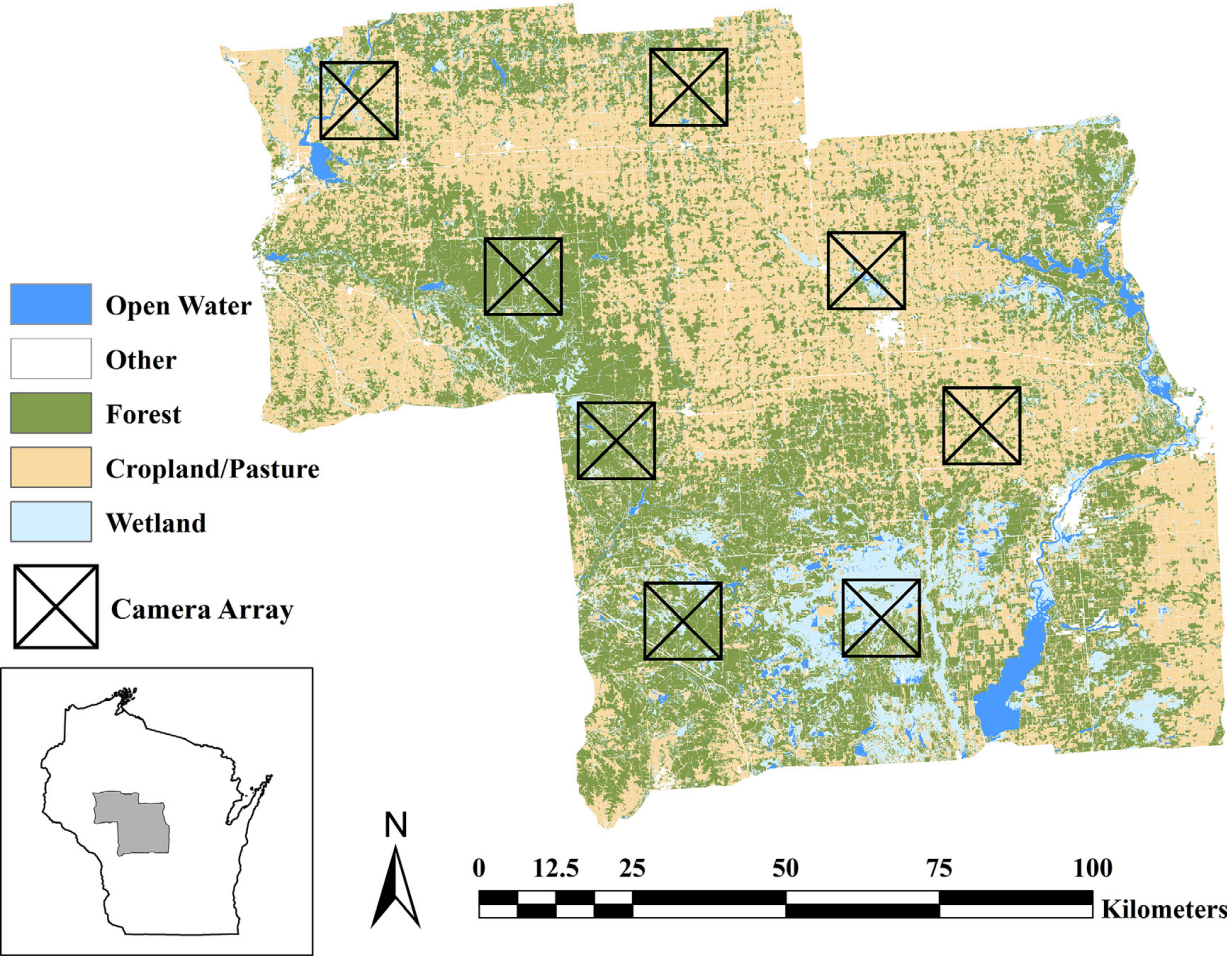
Study area, land cover types, and location of camera arrays used to sample canid carnivore species and prey in central Wisconsin, USA during 2012.

We used data from 281 camera stations placed across the tension zone in central Wisconsin, USA from 21 March 2012 to 9 December 2012. Primary land cover types within the study area included forest (37%), herbaceous mixtures of cropland and pasture (40%), and wetland (9%, Homer et al. 2015). These cover types were generally spatially correlated (Fig. 1). The tension zone (Curtis 1959) represents a boundary between boreal or transitional forest to the north and broadleaf forest to the south, and includes the northern and southern range boundary for many plant species. Human population density ranged from 0.38 to >1900 people per census tract (U.S. Census Bureau 2010).

Cameras were grouped within eight distinct arrays (*n* = 31–36 camera locations per array). Array locations represented a random subsample of 13 potential systematically spaced locations (Clare et al. 2015), and distance between cameras in distinct arrays was >12 km. Each array was active for approximately 42 days, and individual cameras were checked every 14 days. Individual cameras (HC500^®^ or PC800^®^, Reconyx Inc., Holden, WI) were placed along presumed travel paths such as unpaved roads, trails, crop‐edges, or riparian corridors at a height of 50–100 cm. Cameras were set to fire three times per triggering event, with no rest time between triggers. Arrays were designed to be 6 × 6 grids, but exhibited some irregularity dependent upon land accessibility. Mean nearest neighbor distance between cameras in the same array was 1.5 km (SD = 0.46 km).

### Modeling framework

Following previous recent research assessing IGP, we expected that all species were detected imperfectly and used models to correct for false‐negative observations. However, we also believed that species detectability at specific camera locations could be related both to the number of individuals visiting a camera location and the number of visits an individual might make to a camera location. Furthermore, we were specifically interested in decomposing numerical and behavioral associations. To distinguish variance between these processes, a Royle‐Nichols (hereafter RN model) occupancy‐abundance model was used to estimate the detection and abundance of coyotes, foxes, deer, and lagomorphs (Royle and Nichols 2003). Eastern cottontail (*Silvagus floridensianis*) are the most common lagomorph within the study area, and although they co‐occur with snowshoe hare (*Lepus americanus*), the two species could not reliably be distinguished and were pooled. The RN model specifies abundance *N* at site *i* as a latent Poisson random variable with mean *λ*_*i*_: *N*_*i*_ *˜* Poisson (*λ*_*i*_). It defines the probability of detecting an individual animal at site *i* during survey occasion *j* as *r*_*ij*_. Individuals are not distinguished, but the probability of observing the species at a site during an occasion (*p*_*ij*_) is assumed to relate to both abundance and individual detection probability: *p*_*ij*_ = 1 – (1 –* r*_*ij*_)^*Ni*^. Observed presence‐absence *y*_*ij*_ is then a Bernoulli trial with probability *p*_*ij*_: *y*_*ij*_ *˜* Bernoulli (*p*_*ij*_) All species but wolves were modeled within this framework.

Because much of the variance in wolf density relates to pack size, the RN model was not used to consider wolf relative abundance. Instead, we modeled wolf occurrence using the general single‐species occupancy model of MacKenzie et al. (2002), where the latent occurrence state *z* of a given species at site *i* follows a Bernoulli distribution: *z*_*i*_ *˜* Bernoulli (*ψ*_*i*_). In turn, the observed occurrence *y*_*ij*_ of the species at site *i* during visit *j* is considered a function of the probability of detecting a species conditional upon its presence: *y*_*ij*_ *˜ *Bernoulli (*p*_*ij*_ × *z*_*i*_).

We modeled variation in detection or state parameters using covariates on the appropriate link scale, e.g., logit (*ψ*_*i*_)* = β*_0_ *+ β*_1_ *X*_1*i*_ *…β*_*K*_ *X*_*Ki*_. Covariates within the linear predictors of species occurrence or relative abundance included the proportion of cropland and wetland cover extracted from the 2011 National Land Cover Database (Homer et al. 2015) within a 1500 or 250 m radius buffer of the camera location, and the proportion of cropland cover within the minimum convex polygon surrounding any camera array (Table 1). We selected land cover covariates to account for risk and minimize biases associated with unmodeled heterogeneity across a broad suite of species (Miller et al. 2015). Cropland was correlated with forest cover, and we were more interested in the effects of human development than forest types. Wetland cover had some value as a proxy for predation risk: both water and extremely dense woody cover make movement and escape difficult, and major trails running through wetlands were often bound by water on one side or another. Additionally, wetlands have been previous identified as seasonally important habitat for white‐tailed deer (Van Deelen et al. 1998).

**Table 1:** Candidate covariates used to model the distribution and detection probability of a canid carnivore assemblage (wolf, coyote, gray fox, and red fox) and important prey species (white‐tailed deer and lagomorphs) at camera locations in central Wisconsin, 2012

Covariate	Description	Associated Parameters
% Crop (array)	% Cropland within minimum convex polygon of camera array	*λ* a, *ψ* b
% Crop (1.5 km)	% Cropland within 1.5 km radius	*λ*,* ψ*
% Crop (250 m)	% Cropland within 250 m radius	*λ*,* ψ*
% Wetland (1.5 km)	% Wetland within 1.5 km radius	*λ*,* ψ*
% Wetland (250 m)	%Wetland within 250 m radius	*λ*,* ψ*
Open Trail	Binary assignment of trail with no adjacent woody cover	*r* c, *p* d
Large Trail	Binary assignment of trails large enough to fit an automobile	*r*,* p*
Bare Trail	Binary assignment of trails/roads with a bare substrate	*r*,* p*
Crop Edge	Binary assignment for cameras placed along crop edges	*r*,* p*

a Camera‐specific expected abundance.

b Camera‐specific expected occurrence.

c Camera‐specific detection probability for individual animals.

d Camera‐specific detection probability for a species conditional upon its presence.

We modeled variation in detection according to binary designations of specific trail characteristics such as size or substrate (Table 1). Interspecific differences in trail use were expected (Harmsen et al. 2010) and we anticipated that lagomorphs might not trigger a camera as often when moving along larger trails at a greater distance from the camera itself. We included camera placement along crop‐edges as a candidate predictor for detection under the assumption that prey would frequently forage on food crops, and that dependent carnivores might follow prey or exhibit avoidance of anthropogenic activity. Finally, expecting that vegetation cover surrounding the path might be an important security consideration, we predicted detection based upon whether the vegetation surrounding featured woody hiding cover or not. Trail specific factors were coded as binary dummy variables, and continuous covariates were standardized to have mean 0 and standard deviation 0.5.

**Figure 2:**
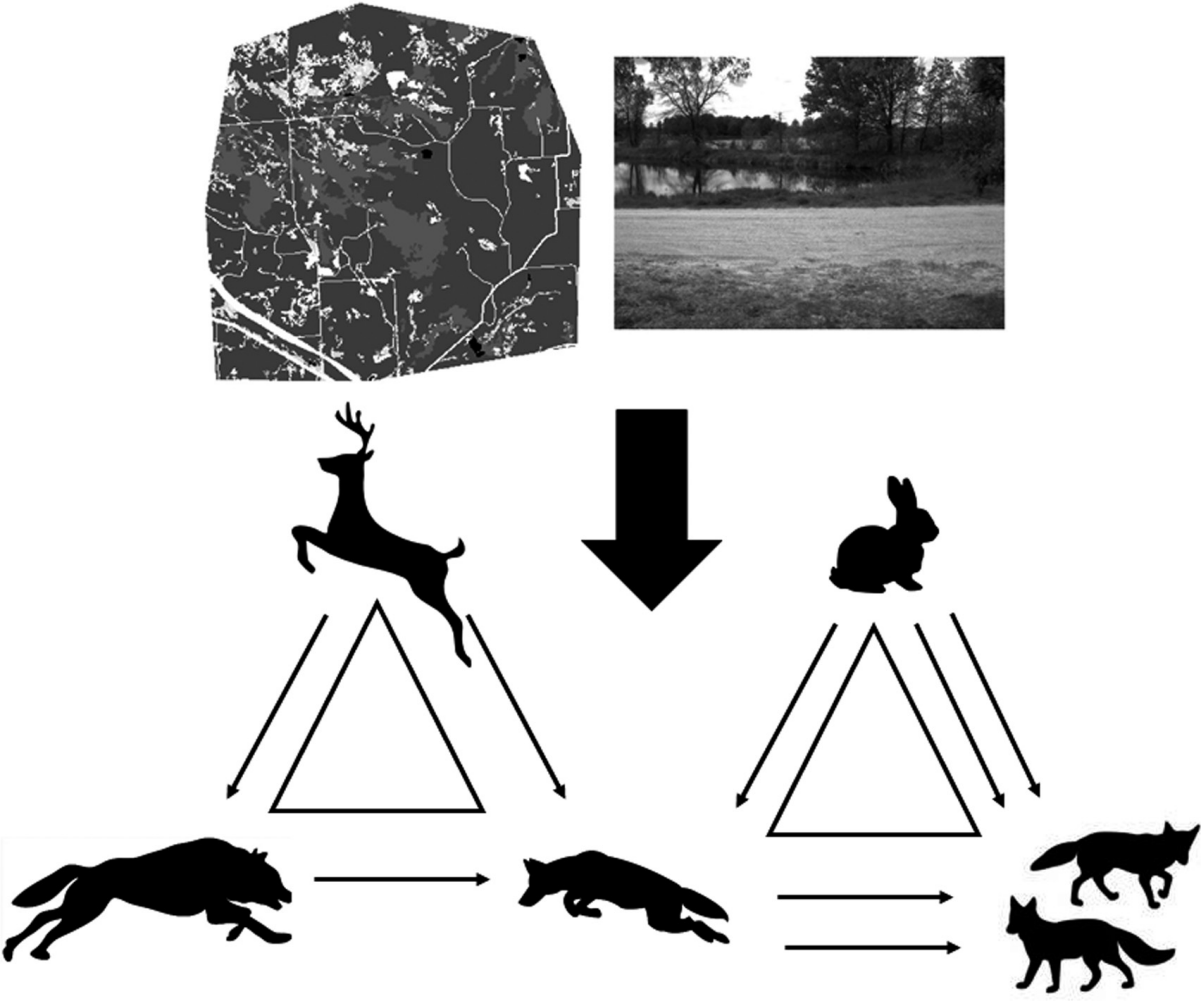
Schematic of species interactions used to model relative abundance or occurrence of canid carnivores and prey in central Wisconsin, USA. All state and detection parameters were functions of land cover and trail characteristics. Arrows extend from independent species to dependent species.

We extended the Waddle et al. (2010) hierarchical formulation of species interactions within an occupancy model to incorporate dependency between the presence or relative abundance of a species and the presence or relative abundance of a prey item or potential intraguild predator. Under species‐interaction occupancy models, presence or detection of one species (*A*) is assumed to be dependent upon the latent state for a second species (*B*). This dependence is modeled as a covariate effect such that for the dependent species A, logit (*ψ*_*A*_) *= β*_0_ *+ β*_1_ *z*_*B*_ or logit (*p*_*A*_) *= β*_0_ *+ β*_1_ *z*_*B,*_ where *β*_1_ reflects the change in the log‐odds of detection or presence of species *A* if species *B* was present. We adapted this to involve variation in *λ* or *r* where necessary: log (*λ*_*A*_) *= β*_0_ *+ β*_1_ *N*_*B*_ or logit (*r*_*A*_) *= β*_0_ *+ β*_1_ *N*_*B*_. Thus, the complete model included abundance and detection submodels for deer and lagomorphs, a wolf detection and occurrence submodel dependent upon deer relative abundance, a coyote detection and abundance submodel dependent both upon deer and lagomorph abundance and wolf occurrence, and a fox detection and abundance submodel dependent upon rabbit and coyote abundance. Additional variation in all detection and state parameters was simultaneously modeled in relation to land cover or trail characteristics (Fig. 2, Appendix S1 contains code).

Within the model, two assumptions were violated. First, there was clearly no population closure within a camera's trigger zone. Thus, *ψ* represents the probability that a typical camera site with specific covariate values would be used by a given species, and *λ* represents the average number of individual animals expected to visit a site with specific covariate values. This interpretation further assumes relatively constant aggregation patterns and relatively constant home range size within species across the study area (Efford and Dawson 2012), but this assumption seems to hold for one species within the study area (Clare et al. 2015). Despite this, associations derived from this model should be more robust than those based on raw counts, and cameras have previously been used to assess species interactions within a hierarchical framework (Steinmetz et al. 2013; Robinson et al. 2014).

The second assumption violated was independence between sites. Intercamera spacing was not sufficient to preclude individual wolves or coyotes from visiting multiple detectors. Although not uncommon for carnivore surveys (Steinmetz et al. 2013), this can potentially provide misleading inferences regarding parameter associations if one site is visited simply due to proximity to another site. Such pseudo‐replication was addressed by treating each camera array as a block and instituting an array‐specific random intercept for all state and detection parameters. This had the added effect of accounting for additional unmodeled variance (e.g., time of year), although improved accuracy comes at the expense of reduced estimate precision for predictors (Kery and Schaub 2012).

### Model fitting, selection, and inference

We used Gibbs Variable Selection (GVS) procedures to select predictors (Dellaportas et al. 2002). GVS associates an inclusion indicator (*w*_*k*_) having a prior distribution of Bernoulli (0.5) to each beta parameter *k* in the model (e.g., *y*_*i*_ = *β*_0_ + *w*_*k*_ **× *β*_*k*_ **× *X*_*ki*_). When *w*_*k*_ **= 0, the beta parameter is considered ‘off’ and posterior samples are drawn from a pseudo‐prior based upon posterior estimates from a pilot run of the full model. When *w*_*k*_ **= 1, posterior beta samples are drawn from Normal (0, Σ_*k*_), where Σ_*k*_ is the prior variance for a beta coefficient. The posterior estimate of each *w*_*k*_ is interpreted as the probability that the best model includes the associated *β*_*k*_, though these probabilities can be very sensitive to the prior specified for Σ_*k*_ (Tenan et al. 2014; Linden and Roloff 2015). We therefore used an approach described by Link and Barker (2006), where each unique linear predictor *θ* (e.g., *p*,* ψ*,* λ*) has a prior variance, *V*_*θ*_, shared between all the *β*_*k*_ for that predictor, such that:

$$\Sigma_{\theta k}=\frac{V_{\theta }}{\sum_{k=1}^{K}w_{\theta k}}$$

The prior for each *V*_*θ*_ is a vague inverse gamma, 1/*V*_*θ*_ ~ Γ(3.2890, 7.8014), which has some desirable properties (Link and Barker 2006). In this way, the prior variance for each *β*_*k*_ depends on how many other regression coefficients are included for the given linear predictor, a restraint that results in shrinkage for the *β*_*k*_ (see Appendix S1 for further detail).

We recognized that associations between species occurrence and relative abundance may have been more evident at broad‐scales rather than at the camera‐level (e.g., Berger and Gese 2007; Newsome and Ripple 2015). To assess broad scale relative abundance or occurrence associations between species, array‐specific parameters equal to the average of abundance (i.e., $\overset{¯}{N}$) or occurrence ($\overset{¯}{z}$) at all cameras within the array were derived during our variable selection model fitting. These averages were considered indices of array‐specific abundance, and correlation coefficients (denoted *ρ* hereafter) were estimated to evaluate interspecific associations in abundance across arrays.

All models were fit using Markov chain Monte Carlo simulation in JAGS (Plummer 2003) via an R interface (R Development Core Team 2015) in library ‘jagsUI’ (Kellner 2015). Code is provided in Appendix S1. Pilot model simulations consisted of 50,000 burn‐in iterations, with 60,000 posterior iterations used for inference (150,000 thinned by 10 across four chains), with a prior for beta parameters set to Uniform (−10, 10). Subsequent variable selection models required longer simulations (300,000 burn‐in iterations, and 200,000 iterations used for inference with the same thinning rate). Convergence was assessed using standard diagnostic tests ($\hat{R}$ < 1.1, Gelman and Rubin 1992) and visual inspection of trace‐plots. Post hoc correlation tests of array‐level abundance were performed using R library ‘BayesianFirstAid’ (Baath 2014).

Inference was made using two statistics. The posterior estimates of *w*_*k*_ were used to gauge the relative importance of candidate predictors. Certainty in the directional effect of covariates conditional upon their inclusion within the model was inferred using credible intervals (CRI). Although confidence in directional effect and inclusion probability are generally related, collinearity between predictors can pose difficulties and result in simplified model structures being selected (Dellaportas et al. 2002). Because our candidate covariates were not orthogonal, considering ${\hat{\beta }}_{k}$|*w*_*k*_ = 1 provides some means to identify potentially influential predictors that may have had low inclusion probability due to collinearity with other important predictors. Due to estimate shrinkage, we consider 85% CRI failing to overlap zero as evidence for confidence in directional effect, although 95% CRI are provided in the supporting information (Tables S1–S4).

## Results

Total sampling effort was 11,984 trap‐nights. Of the species considered, white‐tailed deer (*n* = 278, 98.9%) and coyote (*n* = 146, 51.9%) were detected at the greatest proportion of camera stations and found within each array. Wolves (*n* = 82, 29.2%) and lagomorphs (*n* = 87, 31.0%) were detected at fewer stations, but were also observed within all eight arrays. Red fox (*n* = 42, 14.6%) and gray fox (*n* = 23, 8.1%) were the most infrequently detected organisms within the analysis, and were each observed in four of the eight arrays.

The most important predictors of lagomorph detection were negative associations with bare trails (*w*_*k*_ *= *0.80, ${\hat{\beta }}_{k}$ *= *−0.55*,* 85% CRI *=* −0.92, −0.19) and trails without adjacent woody cover (*w*_*k*_ *= *0.90, ${\hat{\beta }}_{k}$ *= *−0.82*,* 85% CRI *=* −1.33, −0.33). Lagomorph detection was also greater along crop edges and lesser along larger trails (Tables 2 and 3). The strongest predictor of white‐tailed deer individual detection was camera placement along crop edges (*w*_*k*_ *= *0.99*,* ${\hat{\beta }}_{k}$ *= *0.53*,* 85% CRI *=* 0.30, 0.77), and deer detection was lower along paths with bare substrates (*w*_*k*_ *= *0.78*,* ${\hat{\beta }}_{k}$ *= *−0.25*,* 85% CRI *=* −0.39, −0.11). Supported predictors of red fox detection included open trails (*w*_*k*_ *= *0.57*,* ${\hat{\beta }}_{k}$ *= *0.66*,* 85% CRI *=* −0.13, 1.51) and crop edges (*w*_*k*_ *= *0.59*,* ${\hat{\beta }}_{k}$ *= *0.72*,* 85% CRI *=* −0.14, 1.63), but directional effect was uncertain. Wolf and coyote detection probability were both positively associated with well‐worn trails, and coyotes and gray foxes detected more frequently along larger trails (Tables 2 and 3). Other trail factors had limited model support.

**Table 2:** Inclusion probability for candidate predictor covariates of detection probability within a multispecies interaction model based upon camera images in Wisconsin, USA 2012

Species	Predictor			
	Open site	Crop edge	Bare substrate	Large trail
Lagomorph	0.90	0.63	0.80	0.56
Deer	0.42	0.99	0.78	0.16
Red fox	0.57	0.59	0.44	0.44
Gray fox	0.40	0.47	0.40	0.79
Coyote	0.47	0.40	1.00	0.52
Wolf	0.50	0.57	0.89	0.55

**Table 3:** Beta coefficients based upon the posterior mean (standard errors) associated with candidate predictor covariates of detection probability conditional upon covariate inclusion within a multispecies interaction occupancy model based upon camera images in Wisconsin, USA 2012

Species	Covariate			
	Open site	Crop edge	Bare substrate	Large trail
Lagomorph	−0.82 (0.35)^a^	0.40 (0.22)^b^	−0.55 (0.25)^a^	−0.30 (0.16)^b^
Deer	−0.24 (0.20)	0.53 (0.16)^a^	−0.25 (0.10)^a^	0.04 (0.10)
Red fox	0.66 (0.57)	0.72 (0.63)	−0.31 (0.52)	−0.39 (0.48)
Gray fox	0.03 (0.84)	−0.44 (1.09)	0.31 (0.72)	1.50 (0.96)^b^
Coyote	0.35 (0.36)	0.20 (0.37)	0.88 (0.21)^a^	0.36 (0.25)^b^
Wolf	−0.30 (0.57)	−0.28 (0.82)	0.57 (0.23)^a^	0.43 (0.53)

a 95% CRI does not overlap 0.

b 85% CRI does not overlap 0.

Lagomorph abundance was best predicted by a positive association with cropland at a small (250 m) scale and a negative association with wetlands at an intermediate (1.5 km) scale (Tables 4 and 5). White‐tailed deer had negligible association with cropland at any scale, but were less abundant at camera‐locations with more surrounding wetland, particularly within 250 m (*w*_*k*_ *= *0.85*,* ${\hat{\beta }}_{k}$ *= *−0.38*,* 85% CRI *=* −0.58, −0.18). Surrounding wetland cover was the most supported predictor of gray fox abundance, but the association was imprecise (Tables 4 and 5). Similarly, although array‐scale cropland was the most important predictor of red fox abundance, its effect was uncertain (*w*_*k*_ *= *0.67*,* ${\hat{\beta }}_{k}$ *= *0.95*,* 85% CRI *=* −0.68, 2.84). Coyote abundance was generally negatively associated with increased surrounding wetland cover and cropland at the array scale, but (despite limited importance) was positively correlated with surrounding cropland at an intermediate (1.5 km) scale (Tables 4 and 5). In contrast, wolf occurrence was characterized by increased surrounding wetland cover and limited surrounding cropland (Tables 4 and 5).

**Table 4:** Inclusion probability for land‐cover candidate predictor covariates of species occurrence and abundance within a multispecies interaction model based upon camera images in Wisconsin, USA 2012

Species	Covariate				
	% Crop (array)	% Crop (1.5 km)	% Crop (250 m)	% Wetland (1.5 km)	% Wetland (250 m)
Lagomorph	0.36	0.20	0.99	0.77	0.42
Deer	0.19	0.09	0.14	0.23	0.85
Red fox	0.67	0.28	0.47	0.35	0.39
Gray fox	0.49	0.36	0.44	0.61	0.57
Coyote	0.78	0.45	0.37	0.82	0.62
Wolf	0.63	0.31	0.42	0.63	0.82

**Table 5:** Beta coefficients based upon the posterior mean (standard errors) associated with candidate predictor covariates of occurrence or abundance conditional upon covariate inclusion within a multispecies interaction occupancy model based upon camera images in Wisconsin, USA 2012

Species	Covariate				
	% Crop (array)	% Crop (1.5 km)	% Crop (250 m)	% Wetland (1.5 km)	% Wetland (250 m)
Lagomorph	0.08 (0.44)	0.08 (0.16)	0.85 (0.22)^a^	−0.63 (0.31)^a^	−0.19 (0.50)
Deer	0.04 (0.17)	−0.01 (0.06)	−0.05 (0.10)	−0.12 (0.13)	−0.38 (0.14)^a^
Red fox	0.95 (1.24)	−0.10 (0.29)	−0.43 (0.37)	0.09 (0.41)	−0.18 (0.49)
Gray fox	0.13 (0.72)	−0.19 (0.33)	−0.25 (0.49)	−0.63 (0.53)	−0.59 (0.71)
Coyote	−0.72 (0.43)^b^	0.25 (0.18)	0.21 (0.25)	−0.53 (0.24)	−0.52 (0.36)^b^
Wolf	−1.23 (1.22)	0.04 (0.60)	−0.53 (0.74)	1.04 (0.68)^b^	1.49 (0.80)^a^

a 95% CRI does not overlap 0.

b 85% CRI does not overlap 0.

**Figure 3:**
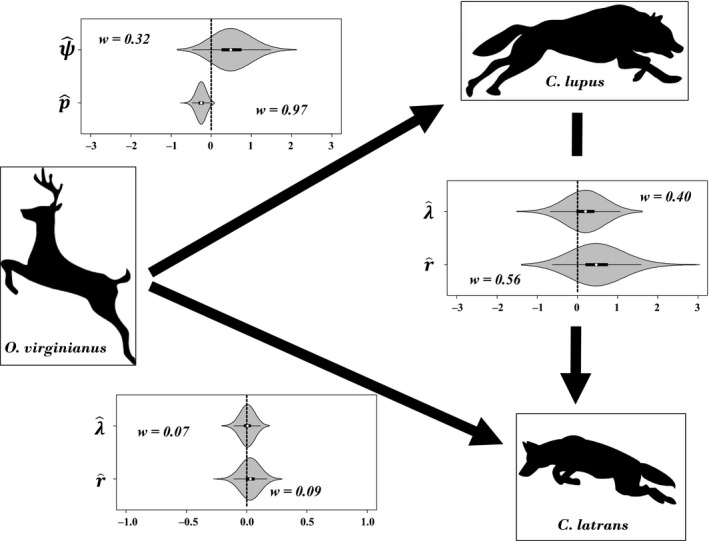
Posterior distributions of coefficients associated with interspecific effects upon camera‐specific relative abundance, occurrence, and detection of white‐tailed deer coyotes, and wolves at camera stations in central Wisconsin, USA. Arrows extend from independent species to dependent species. Box‐plots represent 25%, 50%, and 75% quantiles, with whiskers extending 1.5 times the difference between 75% and 25% quantiles.

In general, there was little model support for most associations between species state parameters at the camera scale. Within the coyote‐wolf‐deer triangular relationship, results suggested that camera‐scale coyote abundance was not dependent upon wolf occurrence (*w*_*k*_ *= *0.40*,* ${\hat{\beta }}_{k}$ *= *0.19*,* 85% CRI *=* −0.29, 0.67), and that wolf occurrence was positively but weakly dependent upon deer abundance (*w*_*k*_ *= *0.32*,* ${\hat{\beta }}_{k}$ *= *0.30*,* 85% CRI *=* 0.02, 0.62). Instead, inter‐specific associations at the camera scale more strongly related to detection. Wolves were detected less frequently at stations where deer more abundant (*w*_*k*_ *= *0.97*,* ${\hat{\beta }}_{k}$ *= *−0.25*,* 85% CRI *=* −0.38, −0.14). Wolf presence was an important but uncertain predictor of coyote detection probability (*w*_*k*_ *= *0.56*,* ${\hat{\beta }}_{k}$ *= *0.50*,* 85% CRI *=* −0.09, 1.15). Neither coyote abundance nor detection had any association with deer abundance (Fig. 3, Tables S1–S4).

**Figure 4:**
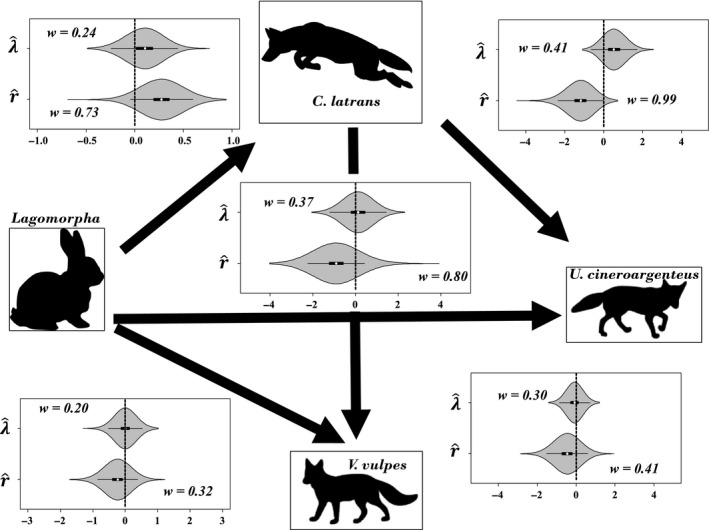
Posterior distributions of coefficients associated with interspecific effects camera‐specific relative abundance and detection of coyotes, red fox, gray fox, and lagomorphs at camera stations in central Wisconsin, USA. Arrows extend from independent species to dependent species. Box‐plots represent 25%, 50%, and 75% quantiles, with whiskers extending 1.5 times the difference between 75% and 25% quantiles.

Within the fox‐coyote‐lagomorph triangular relationship, the associations between state parameters were similarly limited (Fig. 4). Coyotes were detected more frequently where lagomorphs were more abundant (*w*_*k*_ *= *0.73*,* ${\hat{\beta }}_{k}$ *= *0.28*,* 85% CRI *=* 0.11, 0.46), and both red fox (*w*_*k*_ *= *0.80*,* ${\hat{\beta }}_{k}$ *= *−0.88*,* 85% CRI *=* −1.67, −0.06) and gray fox (*w*_*k*_ *= *0.99*,* ${\hat{\beta }}_{k}$ *= *−1.23*,* 85% CRI *=* −1.92, −0.65) were detected less frequently where coyotes were more abundant. There was little relationship between lagomorph abundance and coyote abundance, fox abundance, or fox detection (Fig. 4, Tables S1–S4).

Associations between relative abundance indices at the array scale exhibited different patterns. There was most support for positive correlations between lagomorph abundance and both red fox abundance ($\hat{\rho }$ = 0.55, 85% CRI* *= 0.15, 0.91) and gray fox abundance ($\hat{\rho }$ = 0.75, 85% CRI* *= 0.44, 0.97). There was a negative correlation between lagomorph abundance and coyote abundance ($\hat{\rho }$ = −0.51, 85% CRI* *= −0.89, −0.10). Although wolf and coyote abundance indices were positively associated, there was great uncertainty ($\hat{\rho }$ = 0.39, 85% CRI* *= −0.07, 0.82), and all other array‐scale correlations appeared negligible (Table 6).

**Table 6:** Correlations (85% Credible Intervals) between the average array‐level abundance or occurrence for species within a multispecies interaction model based upon remote camera detections in Wisconsin, USA during 2012. Dashes (–) indicate no correlation coefficient was estimated

	Wolf	Coyote	Red fox	Gray fox
Wolf	–	–	–	–
Coyote	0.39 (−0.07, 0.82)	–	–	–
Red fox	–	−0.28 (−0.74, 0.23)	–	–
Gray fox	–	−0.26 (−0.78, 0.24)	–	–
White‐tailed deer	−0.06 (−0.58, 0.47)	−0.18 (−0.70, 0.34)	–	–
Lagomorph	–	−0.51 (−0.89, −0.10)	0.55 (0.15, 0.91)	0.75 (0.44, 0.97)

## Discussion

Our results are consistent with our primary hypothesis that mesopredator suppression services may be dampened if a shared prey species actively avoids the larger predator in space. This suggests some prey‐based mediation of intra‐guild predation, although as we discuss below, other factors also contribute to mesopredator suppression strength and our analysis represents a simplification of the community. Importantly, our inference is based upon a single hierarchical model that allowed us to distinguish between detection and relative abundance associations, incorporate multiple species interactions simultaneously, and properly account for uncertainty across all processes.

The triangular relationship between coyotes, foxes, and lagomorphs we observed was more consistent with IGP and more consistent with mesopredator suppression. Coyote abundance was not correlated with lagomorph abundance at the camera scale, but individual coyotes were detected at cameras with greater lagomorph abundance more frequently, and the abundance of coyotes and lagomorphs was negatively correlated at the array scale. Generally, lagomorphs are a major component of coyote diets (Bekoff and Gese 2003), but the species has wide dietary breadth and the limited association between their abundance at fine‐scales may reflect nonreliance of coyotes upon lagomorphs (Thompson and Gese 2007). Instead, it appears that areas with high lagomorph density were prone to more frequent usage by individual coyotes. Lagomorphs did not appear to be responding to coyote predation risk by making broad‐scale movements to avoid coyote encounter. Instead, they may have attempted to mitigate risk at fine spatial scales (Bond et al. 2001), as they frequented smaller travel paths used infrequently by coyotes. Lagomorphs were also detected more often in areas with immediate woody cover useful for hiding, but this similarly local strategy is likely more useful for avoiding avian predators (Cox et al. 1997).

In contrast, despite the general commonness of lagomorphs within fox diets across North America (Cypher 2003), neither fox species exhibited any co‐occurrence relationship with lagomorphs at the camera scale. Coyote abundance strongly reduced fox detection, consistent with foxes avoiding coyotes and making use decisions on the basis of safety‐matching rather than resource matching (Rosenheim 2004; Thompson and Gese 2007; Robinson et al. 2014). Although patterns of behavioral avoidance between coyotes and foxes at the camera scale did not manifest as negative population associations at the array scale, time‐series evidence elsewhere (Levi and Wilmers 2012; Newsome and Ripple 2015) suggests that that coyote intimidation of foxes may carry population consequences for the smaller predators (Lima 1998; Preisser et al. 2005; Creel and Christiansen 2008).

Yet the observed negative correlation between coyote and lagomorph abundance at the array‐scale is inconsistent with coyotes practicing resource matching (Rosenheim 2004). This may partially reflect a consumptive effect that theory suggests should be more likely under circumstances of opportunistic predation (Holt 1977; DeCesare et al. 2010). However, we believe that it more likely relates to the very nature of cropland, characterized by substantial herbaceous primary productivity that serves as a positive bottom‐up driver of lagomorph abundance (Hernandez et al. 2011) and substantial human influence that simultaneously creates risky environment for carnivores due to direct exploitation, discussed below. The positive association between foxes and lagomorphs at the array scale may reflect a shared association with landscape productivity, as foxes also feed upon small mammals that are typically more abundant where herbaceous vegetation is more productive (Jedrzejewski and Jedrzejewska 1996), or a shared negative association with other predators more infrequently found in cropland (e.g., Clare et al. 2015).

The triangular relationship between wolves, coyotes, and deer tended to follow predictions of ecology of fear, and there was little evidence of mesopredator suppression. Wolf occurrence was positively associated with deer abundance, but deer abundance and wolf detection probability were negatively correlated, consistent with deer behaving fearfully and regional observations of deer avoiding regions of heavy wolf use (Callan et al. 2013). Wolf occurrence and deer abundance were also respectively greater and lower in wetland cover types that we believed facilitated predation. These observations underscore the importance of deer as a prey item for wolves. They also suggest that wolves and deer made distribution‐scale habitat decisions based upon associated predation risk, a phenomenon observed in other wolf‐prey systems and consistent with fear ecology (Laundre 2010). There were no correlations between coyote and deer parameters, but individual coyotes were detected more frequently when wolves were present, surprisingly suggesting that wolves were not only not intimidating coyotes, but that coyotes may have been selecting for sites of increased wolf use.

However, we acknowledge that agreement between our observations and our expectations does not confirm our proposed mechanism. An obvious alternative mechanism is that larger prey tend to provide a broader carrion resource, and coyotes may use areas of wolf occurrence to take advantage of carrion subsidies (Wilmers et al. 2003). It seems unlikely that coyotes feeding upon smaller prey would leave any carrion for foxes. Our results are also consistent with the hypothesis that dietary overlap influences competitive displacement (Holt and Huxel 2007). Coyote diet regionally generally seems to encompass both wolf and fox food resources. Coyotes may be able to effectively subsist on all fox food items, limiting the low‐resource refugia available to foxes that IGP predicts should increase the likelihood of coexistence, and simultaneously avoid wolf predation by subsisting on prey the larger canid cannot economically exploit.

Our results indicate that top predators may be present without obviously limiting mesopredators. But despite the alternative hypotheses mentioned above, mesopredator suppression has been demonstrated both when mesopredators have limited dietary overlap with larger predators and when shared prey species are large enough to provide a carrion resource (Berger and Gese 2007; Levi and Wilmers 2012; Newsome and Ripple 2015). We discuss additional contextual factors affecting mesopredator suppression below.

Although a neutral or positive wolf‐coyote association contrasts with previous spatial research in the western US (e.g., Berger and Gese 2007), it is consistent with regional longitudinal efforts in similar transitional ecological systems (Levi and Wilmers 2012; Newsome and Ripple 2015). We venture two speculative hypotheses for why wolves may not appear to suppress coyotes in this region. First, cropland may have provided coyotes both a refuge from wolves and access to ample prey, and these regions may have been source populations for dispersers into wolf‐occupied habitat, promoting co‐existence (Amarasekare 2010). Although cropland had either neutral or positive effect on prey abundance, there was some indication that carnivores were negatively associated with cropland at scales related to body size, following predictions of island biogeography (Woodroffe and Ginsberg 1998; Crooks 2002). This implies that carnivore avoidance of anthropogenic activity was driven more by security or survival than resource availability (Valeix et al. 2012), and human caused‐mortality is a previously identified limiting factor for wolf distribution (Stenglein et al. 2015).

A secondary hypothesis is that the region's milder winter weather, lack of elevational heterogeneity, and land cover conversion to promote deer forage may no longer promote seasonal sessility of deer, or that our sampling missed an important competitive window. In response to severe winter weather, deer often migrate to and congregate in dense, closed canopy conifer stands that provide both browse and shelter from snow and cold (Hamerstrom and Blake 1939; Alverson et al. 1988). Coyotes also adjust their space use to deal with snow depth (Dowd et al. 2014). Increased inter‐specific overlap with coyotes and shared prey may allow wolves to exert seasonally dependent suppression (Arjo and Pletscher 1999), and interference competition between the two species is more commonly observed during winter (Merkle et al. 2009). Our sampling may not have captured seasonally mediated competition, and snow‐free months could serve analogously to a temporal refuge for coyotes (Amarasekare 2007).

The concept of a seasonal refuge could be extended to encompass a climatic refuge. Deer yards have historically been associated with conifer wetlands regionally (e.g., Van Deelen et al. 1998). But both reduced winter severity associated with changing climate and row‐crop conversion appear to be delaying or precluding deer migration to seasonal habitats where they are stationary and where wolves have safe access (Sabine et al. 2002; Brinkman et al. 2005). Most evidence for wolf suppression of coyotes comes from systems characterized by harsh winter environments that seasonally confine prey resources (Berger and Gese 2007; Levi and Wilmers 2012; Newsome and Ripple 2015). In the absence of seasonally sessile shared prey, wolves may have limited ability to suppress coyotes.

Finally, carnivore co‐occurrence is likely further dependent upon surrounding landscape structure, with avoidance behaviors stronger in some habitats than in others (Broekhuis et al. 2013). In particular, habitat structure may enhance the ability of smaller carnivores to either perceive or evade larger carnivores (Thompson and Gese 2007; Broekhuis et al. 2013). Although we attempted to correct for distinct habitat preferences when estimating coefficients associated with co‐occurrence, we lacked the data to fit models incorporating fully interactive terms between land cover, trail characteristics, and interspecific dependencies. Thus, some of our estimates of competitive effect may be ambiguous because we may have effectively averaged over a sample with substantial heterogeneity.

It has now been widely demonstrated that top predators have the ability to shape ecosystems (Estes et al. 2011; Ripple et al. 2014). The next logical step toward preserving the influences they exert is to improve prediction of the conditions that facilitate mesopredator or prey regulation. Ultimately, our results suggest that specific aspects of prey life history may be useful for predicting what ecological effects or benefits top carnivores provide. But they also indicate that these effects are highly vulnerable to anthropogenic influence such as habitat conversion, climate change, and direct exploitation.

## Conflict of Interest

None declared.

## Supporting information


**Appendix S1.** Code used to fit occupancy/abundance models.
**Table S1.** 95% Credible intervals associated with occurrence or abundance beta parameters conditional upon model inclusion.
**Table S2.** 85% Credible intervals associated with occurrence or abundance beta parameters conditional upon model inclusion.
**Table S3.** 95% Credible intervals associated with detection beta parameters conditional upon model inclusion.
**Table S4.** 85% Credible intervals associated with detection beta parameters conditional upon model inclusion.
