# Supplementary material for: Do the antipredator strategies of shared prey mediate intraguild predation and mesopredator suppression?
Source: Ecol Evol. 2016 May 10;6(12):3884–97. doi: 10.1002/ece3.2170 (PMC4865477; doi:10.1002/ece3.2170)
Supplement: Supplementary file 1 — Appendix S1. Code used to fit occupancy/abundance models. Table S1. 95% Credible intervals associated with occurrence or abundance beta parameters conditional upon model inclusion. Table S2. 85% Credible intervals associated with occurrence or abundance beta parameters conditional upon model inclusion. Table S3. 95% Credible intervals associated with detection beta parameters conditional upon model inclusion. Table S4. 85% Credible intervals associated with detection beta parameters conditional upon model inclusion. [file ECE3-6-3884-s001.docx]

Appendix S1. Code used to fit occupancy/abundance models

model {

##############################Priors########################################

#####Prey

for (prey in 1:2){#1 lagomorph, 2 deer

mu.p.prey[prey]~dunif(0, 1)

mu.beta.prey[prey]<-log(mu.p.prey[prey])-log(1-mu.p.prey[prey])

sig.p.prey[prey]~dunif(0, 50)

tau.p.prey[prey]<-1/sig.p.prey[prey]^2

mu.lam.prey[prey]~dunif(0, 25)

alpha.lam.prey[prey]<-log(mu.lam.prey[prey])

sig.lam.prey[prey]~dunif(0, 50)

tau.lam.prey[prey]<-1/sig.lam.prey[prey]^2

for (g in 1:8){

a0.prey[prey, g]~dnorm(alpha.lam.prey[prey], tau.lam.prey[prey])T(-10, 10)

b0.prey[prey, g]~dnorm(mu.beta.prey[prey], tau.p.prey[prey])T(-10, 10)

}

###### Note, tau.prey.beta[]/tau.fox.beta[], etc. is the posterior precision estimate from

###### the full pilot run; while mu.prey.beta[]/mu.fox.beta[] etc. is the posterior mean from the

###### the full pilot run

for (beta in 1:9){

w.prey[beta,prey]~dbern(.5)

beta.mu.prey[beta,prey]<-(1-w.prey[beta,prey])*mu.prey.beta[beta,prey]

}

for(p in 1:2){ #lam and r

tau.V.prey[p,prey] ~ dgamma(3.28,7.80)

}

sumw.prey[1,prey] <- sum(w.prey[1:5,prey])+1 #lam; note that the +1 accounts for the intercept and prevents an

#####impossible denominator below

for (beta in 1:5){

beta.tau.prey[beta,prey]<-w.prey[beta, prey]*(1/((1/tau.V.prey[1,prey])/sumw.prey[1,prey])) +

(1-w.prey[beta,prey])*tau.prey.beta[beta,prey]

b.prey[beta,prey]~dnorm(beta.mu.prey[beta,prey], beta.tau.prey[beta,prey])

}

sumw.prey[2,prey] <- sum(w.prey[6:9,prey])+1 #r

for (beta in 6:9){

beta.tau.prey[beta, prey]<-w.prey[beta, prey]*(1/((1/tau.V.prey[2,prey])/sumw.prey[2,prey])) +

(1-w.prey[beta,prey])*tau.prey.beta[beta,prey]

b.prey[beta,prey]~dnorm(beta.mu.prey[beta,prey], beta.tau.prey[beta,prey])

}

}#prey

######coyote priors

mu.p.coy~dunif(0, 1)

mu.beta.coy<-log(mu.p.coy)-log(1-mu.p.coy)

sig.p.coy~dunif(0, 50)

tau.p.coy<-1/sig.p.coy^2

mu.lam.coy~dunif(0, 10)

alpha.lam.coy<-log(mu.lam.coy)

sig.lam.coy~dunif(0, 50)

tau.lam.coy<-1/sig.lam.coy^2

for (g in 1:8){

a0.coy[g]~dnorm(alpha.lam.coy, tau.lam.coy)T(-10, 10)

b0.coy[g]~dnorm(mu.beta.coy, tau.p.coy)T(-10, 10)

}

#------GVS betas------------

for (beta in 1:15){

w.coy[beta]~dbern(.5)

beta.mu.coy[beta]<-(1-w.coy[beta])*mu.coy.beta[beta]

}

for(p in 1:2){ #lam and r

tau.V.coy[p] ~ dgamma(3.28,7.80)

}

sumw.coy[1] <- sum(w.coy[1:8])+1 #lam

for (beta in 1:8){

beta.tau.coy[beta]<-w.coy[beta]*(1/((1/tau.V.coy[1])/sumw.coy[1])) +

(1-w.coy[beta])*tau.coy.beta[beta]

b.coy[beta]~dnorm(beta.mu.coy[beta], beta.tau.coy[beta])

}

sumw.coy[2] <- sum(w.coy[9:15])+1 #r

for (beta in 9:15){

beta.tau.coy[beta]<-w.coy[beta]*(1/((1/tau.V.coy[2])/sumw.coy[2])) +

(1-w.coy[beta])*tau.coy.beta[beta]

b.coy[beta]~dnorm(beta.mu.coy[beta], beta.tau.coy[beta])

}

#####fox priors

for(fox in 1:2){#1<-red fox, 2<-gray fox

mu.p.fox[fox]~dunif(0, 1)

mu.beta.fox[fox]<-log(mu.p.fox[fox])-log(1-mu.p.fox[fox])

sig.p.fox[fox]~dunif(0, 50)

tau.p.fox[fox]<-1/sig.p.fox[fox]^2

mu.lam.fox[fox]~dunif(0, 10)

alpha.lam.fox[fox]<-log(mu.lam.fox[fox])

sig.lam.fox[fox]~dunif(0, 50)

tau.lam.fox[fox]<-1/sig.lam.fox[fox]^2

for (g in 1:8){

a0.fox[fox, g]~dnorm(alpha.lam.fox[fox], tau.lam.fox[fox])T(-10, 10)

b0.fox[fox, g]~dnorm(mu.beta.fox[fox], tau.p.fox[fox])T(-10, 10)

}

#------GVS betas------------

for (beta in 1:13){

w.fox[beta,fox]~dbern(.5)

beta.mu.fox[beta,fox]<-(1-w.fox[beta,fox])*mu.fox.beta[beta,fox]

}

for(p in 1:2){ #lam and r

tau.V.fox[p,fox] ~ dgamma(3.28,7.80)

}

sumw.fox[1,fox] <- sum(w.fox[1:7,fox])+1 #lam

for (beta in 1:7){

beta.tau.fox[beta, fox]<-w.fox[beta, fox]*(1/((1/tau.V.fox[1,fox])/sumw.fox[1,fox])) +

(1-w.fox[beta,fox])*tau.fox.beta[beta,fox]

b.fox[beta, fox]~dnorm(beta.mu.fox[beta,fox], beta.tau.fox[beta,fox])

}

sumw.fox[2,fox] <- sum(w.fox[8:13,fox])+1 #r

for (beta in 8:13){

beta.tau.fox[beta, fox]<-w.fox[beta, fox]*(1/((1/tau.V.fox[2,fox])/sumw.fox[2,fox])) +

(1-w.fox[beta,fox])*tau.fox.beta[beta,fox]

b.fox[beta, fox]~dnorm(beta.mu.fox[beta,fox], beta.tau.fox[beta,fox])

}

}

#####wolf priors

mu.p.wolf~dunif(0, 1)

mu.psi.wolf~dunif(0,1)

mu.alpha.wolf<-log(mu.psi.wolf)-log(1-mu.psi.wolf)

mu.beta.wolf<-log(mu.p.wolf)-log(1-mu.p.wolf)

sig.p.wolf~dunif(0, 50)

sig.psi.wolf~dunif(0, 50)

tau.p.wolf<-1/sig.p.wolf^2

tau.psi.wolf<-1/sig.psi.wolf^2

for (g in 1:8){

a0.wolf[g]~dnorm(mu.alpha.wolf, tau.psi.wolf)T(-10, 10)

b0.wolf[g]~dnorm(mu.beta.wolf, tau.p.wolf)T(-10, 10)

}

#------GVS betas------------

for (beta in 1:11){

w.wolf[beta]~dbern(.5)

beta.mu.wolf[beta]<-(1-w.wolf[beta])*mu.wolf.beta[beta]

}

for(p in 1:2){ #psi and p

tau.V.wolf[p] ~ dgamma(3.28,7.80)

}

sumw.wolf[1] <- sum(w.wolf[1:6])+1 #psi

for (beta in 1:6){

beta.tau.wolf[beta]<-w.wolf[beta]*(1/((1/tau.V.wolf[1])/sumw.wolf[1])) +

(1-w.wolf[beta])*tau.wolf.beta[beta]

b.wolf[beta]~dnorm(beta.mu.wolf[beta], beta.tau.wolf[beta])

}

sumw.wolf[2] <- sum(w.wolf[7:11])+1 #p

for (beta in 7:11){

beta.tau.wolf[beta]<-w.wolf[beta]*(1/((1/tau.V.wolf[2])/sumw.wolf[2])) +

(1-w.wolf[beta])*tau.wolf.beta[beta]

b.wolf[beta]~dnorm(beta.mu.wolf[beta], beta.tau.wolf[beta])

}

##############################Likelihood########################################

for (j in 1:281) {

for (prey in 1:2){

N.prey[prey,j]~dpois(lambda.prey[prey, j])####prey abundance likelihood

log(lambda.prey[prey, j])<-a0.prey[prey, grid[j]]+w.prey[1, prey]*b.prey[1, prey]*Crop.grid[j]+w.prey[2,

prey]*b.prey[2, prey]*cropland[j]+w.prey[3, prey]*b.prey[3, prey]*cropsmall[j]+w.prey[4, prey]*b.prey[4,

prey]*Wetland[j]+w.prey[5, prey]*b.prey[5, prey]*wetsmall[j]

logit(r.prey[prey, j])<-b0.prey[prey, grid[j]]+w.prey[6,prey]*b.prey[6, prey]*open[j]+w.prey[7, prey]*b.prey[7,

prey]*crop.edge[j]+w.prey[8, prey]*b.prey[8, prey]*bare[j]+w.prey[9, prey]*b.prey[9, prey]*NV[j]

p.prey[prey, j]<-1-(1-r.prey[prey, j])^N.prey[prey, j]

y.prey[prey, j]~dbin(p.prey[prey, j], numdays[j])

}

for (fox in 1:2){

N.fox[fox,j]~dpois(lambda.fox[fox, j])####fox abundance likelihood

log(lambda.fox[fox, j])<-a0.fox[fox, grid[j]]+w.fox[1, fox]*b.fox[1, fox]*Crop.grid[j]+w.fox[2, fox]*b.fox[2,

fox]*cropland[j]+w.fox[3, fox]*b.fox[3, fox]*cropsmall[j]+w.fox[4, fox]*b.fox[4, fox]*Wetland[j]+w.fox[5,

fox]*b.fox[5, fox]*wetsmall[j]+w.fox[6, fox]*b.fox[6, fox]*N.prey[1, j]+w.fox[7, fox]*b.fox[7, fox]*N.coy[j]

logit(r.fox[fox, j])<-b0.fox[fox, grid[j]]+w.fox[8,fox]*b.fox[8, fox]*open[j]+w.fox[9, fox]*b.fox[9,

fox]*crop.edge[j]+w.fox[10, fox]*b.fox[10, fox]*bare[j]+w.fox[11, fox]*b.fox[11, fox]*NV[j]+w.fox[12,

fox]*b.fox[12, fox]*N.prey[1, j]+w.fox[13, fox]*b.fox[13, fox]*N.coy[j]

p.fox[fox, j]<-1-(1-r.fox[fox, j])^N.fox[fox, j]

y.fox[fox, j]~dbin(p.fox[fox, j], numweeks[j])

}

N.coy[j]~dpois(lambda.coy[j])####coyote abundance likelihood

log(lambda.coy[j])<-a0.coy[grid[j]]+w.coy[1]*b.coy[1]*Crop.grid[j]+w.coy[2]*b.coy[2]*cropland[j]

+w.coy[3]*b.coy[3]*cropsmall[j]+w.coy[4]*b.coy[4]*Wetland[j]+w.coy[5]*b.coy[5]*wetsmall[j]

+w.coy[6]*b.coy[6]*N.prey[1, j]+w.coy[7]*b.coy[7]*N.prey[2, j]+w.coy[8]*b.coy[8]*z.wolf[j]

logit(r.coy[j])<-b0.coy[grid[j]]+w.coy[9]*b.coy[9]*open[j]+w.coy[10]*b.coy[10]*crop.edge[j]

+w.coy[11]*b.coy[11]*bare[j]+w.coy[12]*b.coy[12]*NV[j]+w.coy[13]*b.coy[13]*N.prey[1,j]+w.coy[14]*b.coy[14]*N.prey[2, j]+w.coy[15]*b.coy[15]*z.wolf[j]

p.coy[j]<-1-(1-r.coy[j])^N.coy[j]

y.coy[j]~dbin(p.coy[j], numweeks[j])

####Wolf occupancy likelihood

z.wolf[j]~dbern(psi.wolf[j])

logit(psi.wolf[j])<-a0.wolf[grid[j]]+w.wolf[1]*b.wolf[1]*Crop.grid[j]+w.wolf[2]*b.wolf[2]*cropland[j]

+w.wolf[3]*b.wolf[3]*cropsmall[j]+w.wolf[4]*b.wolf[4]*Wetland[j]+w.wolf[5]*b.wolf[5]*wetsmall[j]

+w.wolf[6]*b.wolf[6]*N.prey[2, j]

logit(p.wolf[j])<-b0.wolf[grid[j]]+w.wolf[7]*b.wolf[7]*open[j]+w.wolf[8]*b.wolf[8]*crop.edge[j]

+w.wolf[9]*b.wolf[9]*bare[j]+w.wolf[10]*b.wolf[10]*NV[j]+w.wolf[11]*b.wolf[11]*N.prey[2, j]

p.eff.wolf[j]<-z.wolf[j]*p.wolf[j]

y.wolf[j]~dbin(p.eff.wolf[j], numweeks[j])

}

#####Derived parameters, array level abundance indices

mean.coy[1]<-mean(N.coy[1:36])

mean.coy[2]<-mean(N.coy[37:72])

mean.coy[3]<-mean(N.coy[73:108])

mean.coy[4]<-mean(N.coy[109:144])

mean.coy[5]<-mean(N.coy[145:178])

mean.coy[6]<-mean(N.coy[179:209])

mean.coy[7]<-mean(N.coy[210:245])

mean.coy[8]<-mean(N.coy[246:281])

mean.wolf[1]<-mean(z.wolf[1:36])

mean.wolf[2]<-mean(z.wolf[37:72])

mean.wolf[3]<-mean(z.wolf[73:108])

mean.wolf[4]<-mean(z.wolf[109:144])

mean.wolf[5]<-mean(z.wolf[145:178])

mean.wolf[6]<-mean(z.wolf[179:209])

mean.wolf[7]<-mean(z.wolf[210:245])

mean.wolf[8]<-mean(z.wolf[246:281])

for (fox in 1:2){

mean.fox[1, fox]<-mean(N.fox[fox, 1:36])

mean.fox[2, fox]<-mean(N.fox[fox, 37:72])

mean.fox[3, fox]<-mean(N.fox[fox, 73:108])

mean.fox[4, fox]<-mean(N.fox[fox, 109:144])

mean.fox[5, fox]<-mean(N.fox[fox, 145:178])

mean.fox[6, fox]<-mean(N.fox[fox, 179:209])

mean.fox[7, fox]<-mean(N.fox[fox, 210:245])

mean.fox[8, fox]<-mean(N.fox[fox, 246:281])

}

for (prey in 1:2){

mean.prey[1, prey]<-mean(N.prey[prey, 1:36])

mean.prey[2, prey]<-mean(N.prey[prey, 37:72])

mean.prey[3, prey]<-mean(N.prey[prey, 73:108])

mean.prey[4, prey]<-mean(N.prey[prey, 109:144])

mean.prey[5, prey]<-mean(N.prey[prey, 145:178])

mean.prey[6, prey]<-mean(N.prey[prey, 179:209])

mean.prey[7, prey]<-mean(N.prey[prey, 210:245])

mean.prey[8, prey]<-mean(N.prey[prey, 246:281])

}

}

Table S1. 95% Credible intervals associated with occurrence or abundance beta parameters conditional upon model inclusion.

|  | Covariate | | | | | | | | | |
| --- | --- | --- | --- | --- | --- | --- | --- | --- | --- | --- |
| Species | %Crop (array) | % Crop (1.5 km) | % Crop (250 m) | % Wetland (1.5 km) | % Wetland (250 m) | Lagomorph abundance | Deer abundance | Coyote abundance | Wolf Presence |  |
| Lagomorph | -0.79, 0.97 | -0.24, 0.40 | 0.40, 1.29 | -1.27, -0.04 | -1.18, 0.74 | - | - | - | - |  |
| Deer | -0.31, 0.36 | -0.14, 0.11 | -0.25, 0.16 | -0.35, 0.13 | -0.66, -0.10 | - | - | - | - |  |
| Red fox | -1.25, 3.46 | -0.65, 0.49 | -1.18, 0.29 | -0.77, 0.87 | -1.23, 0.75 | -0.41, 0.46 | - | -0.86, 0.84 | - |  |
| Gray fox | -1.27, 1.56 | -0.87, 0.44 | -1.25, 0.69 | -1.76, 0.32 | -2.18, 0.62 | -0.69, 0.54 | - | -0.33, 0.99 | - |  |
| Coyote | -1.59, 0.12 | -0.10, 0.59 | -0.29, 0.70 | -0.99, -0.07 | -1.23, 0.16 | -0.16, 0.33 | -0.08, 0.08 | - | -0.51, 0.85 |  |
| Wolf | -3.67, 1.04 | -1.07, 1.29 | -2.05, 0.88 | -0.16, 2.50 | 0.05, 3.21 | - | -0.08, 0.78 | - | - |  |

Table S2. 85% Credible intervals associated with occurrence or abundance beta parameters conditional upon model inclusion.

|  | Covariate | | | | | | | | | |
| --- | --- | --- | --- | --- | --- | --- | --- | --- | --- | --- |
| Species | %Crop (array) | % Crop (1.5 km) | % Crop (250 m) | % Wetland (1.5 km) | % Wetland (250 m) | Lagomorph abundance | Deer abundance | Coyote abundance | Wolf Presence |  |
| Lagomorph | -0.53, 0.70 | -0.16, 0.31 | 0.53, 1.17 | -1.09, -0.19 | -0.91, 0.51 | - | - | - | - |  |
| Deer | -0.20, 0.27 | -0.11, 0.08 | -0.20, 0.10 | -0.30, 0.07 | -0.58, -0.18 | - | - | - | - |  |
| Red fox | -0.68, 2.84 | -0.50, 0.31 | -0.97, 0.10 | -0.52, 0.66 | -0.91, 0.51 | -0.29, 0.30 | - | -0.63, 0.61 | - |  |
| Gray fox | -0.84, 1.11 | -0.67, 0.27 | -0.96, 0.44 | -1.41, 0.08 | -1.59, 0.31 | -0.51, 0.35 | - | -0.16, 0.78 | - |  |
| Coyote | -1.34, -0.13 | -0.01, 0.50 | -0.15, 0.57 | -0.87, -0.19 | -1.05, -0.01 | -0.10, 0.27 | -0.06, 0.06 | - | -0.29, 0.67 |  |
| Wolf | -3.00, 0.45 | -0.78, 0.92 | -1.60, 0.50 | 0.13, 2.03 | 0.40, 2.68 | - | 0.02, 0.62 | - | - |  |

Table S3. 95% Credible intervals associated with detection beta parameters conditional upon model inclusion.

|  | Predictor | | | | | | | | |
| --- | --- | --- | --- | --- | --- | --- | --- | --- | --- |
| Species | | Open Site | Crop Edge | Bare Substrate | Large Trail | Lagomorph Abundance | Deer Abundance | Coyote Abundance | Wolf Presence |
| Lagomorph | | -1.51, -0.16 | -0.03, 0.82 | -1.06, -0.07 | -0.61, 0.02 | - | - | - | - |
| Deer | | -0.62, 0.18 | 0.22, 0.85 | -0.44, -0.06 | -0.17, 0.23 | - | - | - | - |
| Red fox | | -0.40, 1.87 | -0.46, 2.02 | -1.29, 0.80 | -1.38, 0.53 | -0.77, 0.22 | - | -2.03, 0.46 | - |
| Gray fox | | -1.54, 1.82 | -2.84, 1.57 | -1.13, 1.74 | -0.19, 3.56 | -1.33, 0.32 | - | -2.27, -0.49 | - |
| Coyote | | -0.35, 1.07 | -0.53, 0.94 | 0.46, 1.29 | -0.13, 0.86 | 0.04, 0.53 | -0.07, 0.14 | - | -0.31, 1.50 |
| Wolf | | -1.49, 0.76 | -2.00, 1.27 | 0.12, 1.02 | -0.62, 1.49 | - | -0.43, -0.10 | - | - |

Table S4. 85% Credible intervals associated with detection beta parameters conditional upon model inclusion.

|  | Predictor | | | | | | | | |
| --- | --- | --- | --- | --- | --- | --- | --- | --- | --- |
| Species | | Open Site | Crop Edge | Bare Substrate | Large Trail | Lagomorph Abundance | Deer Abundance | Coyote Abundance | Wolf Presence |
| Lagomorph | | -1.33, -0.33 | 0.09, 0.71 | -0.92, -0.19 | -0.53, -0.07 | - | - | - | - |
| Deer | | -0.52, 0.06 | 0.30, 0.76 | -0.39, -0.11 | -0.11, 0.18 | - | - | - | - |
| Red fox | | -0.13, 1.51 | -0.14, 1.63 | -1.03, 0.45 | -1.08, 0.28 | -0.60, 0.09 | - | -1.67, -0.06 | - |
| Gray fox | | -1.11, 1.23 | -2.00, 0.99 | -0.72, 1.33 | 0.21, 2.93 | -1.06, 0.10 | - | -1.92, -0.65 | - |
| Coyote | | -0.16, 0.87 | -0.33, 0.74 | 0.58, 1.18 | 0.00, 0.73 | 0.11, 0.46 | -0.04, 0.11 | - | -0.09, 1.15 |
| Wolf | | -1.13, 0.49 | -1.44, 0.82 | 0.24, 0.90 | -0.33, 1.20 | - | -0.38, -0.14 | - | - |
